# Wildlife and Antibiotic Resistance

**DOI:** 10.3389/fcimb.2022.873989

**Published:** 2022-05-11

**Authors:** Pablo Laborda, Fernando Sanz-García, Luz Edith Ochoa-Sánchez, Teresa Gil-Gil, Sara Hernando-Amado, José Luis Martínez

**Affiliations:** ^1^Centro Nacional de Biotecnología, Consejo Superior de Investigaciones Científicas (CSIC), Madrid, Spain; ^2^Programa de Doctorado en Biociencias Moleculares, Universidad Autónoma de Madrid, Madrid, Spain; ^3^Departamento de Microbiología, Medicina Preventiva y Salud Pública, Universidad de Zaragoza, Zaragoza, Spain

**Keywords:** One Health, Global Health, antibiotic resistance, wild life, infection

## Abstract

Antibiotic resistance is a major human health problem. While health care facilities are main contributors to the emergence, evolution and spread of antibiotic resistance, other ecosystems are involved in such dissemination. Wastewater, farm animals and pets have been considered important contributors to the development of antibiotic resistance. Herein, we review the impact of wildlife in such problem. Current evidence supports that the presence of antibiotic resistance genes and/or antibiotic resistant bacteria in wild animals is a sign of anthropic pollution more than of selection of resistance. However, once antibiotic resistance is present in the wild, wildlife can contribute to its transmission across different ecosystems. Further, the finding that antibiotic resistance genes, currently causing problems at hospitals, might spread through horizontal gene transfer among the bacteria present in the microbiomes of ubiquitous animals as cockroaches, fleas or rats, supports the possibility that these organisms might be bioreactors for the horizontal transfer of antibiotic resistance genes among human pathogens. The contribution of wildlife in the spread of antibiotic resistance among different hosts and ecosystems occurs at two levels. Firstly, in the case of non-migrating animals, the transfer will take place locally; a One Health problem. Paradigmatic examples are the above mentioned animals that cohabit with humans and can be reservoirs and vehicles for antibiotic resistance dissemination. Secondly, migrating animals, such as gulls, fishes or turtles may participate in the dissemination of antibiotic resistance across different geographic areas, even between different continents, which constitutes a Global Health issue.

## Introduction

Antibiotic resistance (AR) is nowadays one of the main human health problems (Antimicrobial Resistance Collaborators, 2022). Its development has two interconnected aspects: emergence and transmission (Martinez and Baquero, 2014). Emergence is linked to selection and hence it depends on the selective pressure, mainly antibiotics (Martinez et al., 2007; Martinez et al., 2011). For this reason, over decades, judicious use of antibiotics -mainly restricting said use- has been proposed as the cornerstone to tackle AR. A second aspect of AR is transmission (Baquero, 2017; Martínez, 2018; Baquero et al., 2019). Differing with emergence, transmission does not necessarily require antibiotic selective pressure to occur. In support of this concept, it has been described that travellers from countries with a high prevalence of some antibiotic resistance genes (ARGs) frequently acquire these genes, despite not presenting symptoms of infections and not having received antibiotic therapy (Murray et al., 1990; Lane et al., 2016; Reuland et al., 2016). This means that, while restricting the use of antibiotics is valuable, this measure is not enough to tackle the AR problem since it does not always impact on its transmission.

**Figure 1:**
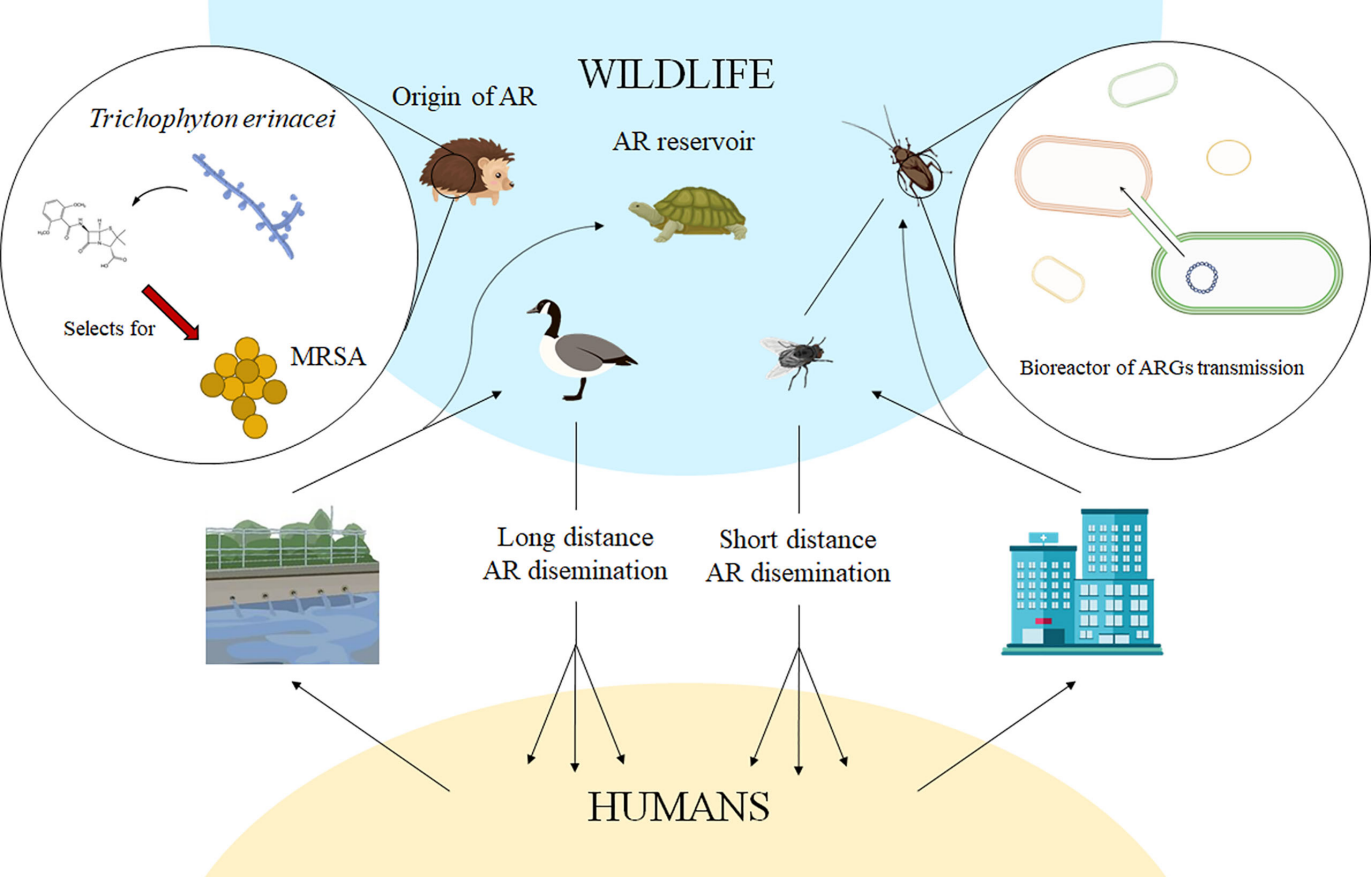
Contribution of wildlife to antibiotic resistance. When talking of the problem of antibiotic resistance, it is important to highlight that it refers to antibiotic resistant bacteria or antibiotic resistance genes that are a problem for human health, not just to the plethora of intrinsically resistant bacteria or resistance genes present in any ecosystem but not involved in human’s infections. Current evidence supports that human antibiotic resistant pathogens and the resistance genes they harbour are found in wild animals, which are hence a reservoir of antibiotic resistance. In the case of migratory animals, as gulls, they might contribute to long-distance dissemination of antibiotic resistance, while non-migrating animals, as flies or cockroaches, may have a role in said dissemination at shorter distances. The finding that antibiotic resistance genes can disseminate across bacteria present in the microbiota of insects, as cockroaches, which have a tight contact with humans, supports that these organisms might act as bioreactors mediating the spread of antibiotic resistance among bacterial pathogens. Besides their involvement in the dissemination of antibiotic resistance, recent findings support that wild animals might have a role in the origin of antibiotic resistance. This is the situation of methicillin resistant *S. aureus* (MRSA) that might have been selected in hedgehogs, long before the therapeutic use of antibiotics, as a response to the presence of β-lactam-producing microorganisms in the microbiome of this wild mammal. AR, antibiotic resistance.

Being transmission an important element in AR spread, knowing the transmission routes is important to tackle the AR problem. In that sense, it has become evident that nearly every ecosystem in the biosphere can participate, at different degree, in the origin, evolution and spread of AR (Aminov, 2009; Martinez et al., 2009; Davies and Davies, 2010; Wellington et al., 2013; Martinez and Baquero, 2014; Berendonk et al., 2015; Martinez et al., 2015a; Larsson et al., 2018). Understanding the AR problem, and most particularly AR transmission, requires two intertwined approaches: One Health and Global Health (Hernando-Amado et al., 2020). One Health studies the role that different, interconnected ecosystems (i. e: hospitals, water bodies, food, pets or farms) may have on the emergence and dissemination of AR (Robinson et al., 2016; Hernando-Amado et al., 2019), while Global Health studies how the emergence of AR in a given geographic area can impact other regions worldwide (Bush et al., 2011; Berndtson, 2020). Several authoritative works and reviews have been published on the role of farming and water in the emergence and spread of AR with relevance for human health (Berendonk et al., 2015). However, while it is known that wildlife can also impact AR, mainly through the transmission of antibiotic resistant microorganisms (**Figure 1**), there are fewer studies in this regard (Dolejska and Literak, 2019; Ramey and Ahlstrom, 2020). Herein, we review how wildlife can globally impact the AR problem.

## Wildlife as a Marker of Antibiotic Resistance Pollution and as an Antibiotic Resistance Reservoir

As stated above, AR emergence occurs under selection, mainly by antibiotics, although other elements, like heavy metals or biocides, may also select for AR. This means that human-linked habitats -and not natural, non-heavily polluted habitats, as wildlife- are the main places in which AR selection occurs. For this reason, when clinically-relevant ARGs and antibiotic-resistant bacteria (ARB) are found in wild animals -not receiving antibiotics- this should be considered as a marker of AR pollution (Martinez, 2009; Martinez and Olivares, 2012), more than a sign of AR selection. Supporting this concept, the study of Australian sea turtles has shown that their microbiome can contain antibiotic resistant *Enterobacteriaceae*, including common human commensals/pathogens such as *Klebsiella*, *Citrobacter* or *Escherichia* and that AR was lower in sampling locations further away from urban areas (Ahasan et al., 2017). Further, the study of *Iguana delicatissima* from the Lesser Antilles also showed that these animals harbour antibiotic resistant *Enterobacteriaceae* and that multi-drug resistant strains were more frequently found in animals from the more anthropized sites. The authors concluded that human-associated bacteria, as well as their ARGs, were mainly acquired by wildlife as a consequence of anthropic ARB pollution of the ecosystems they inhabit (Di Lallo et al., 2021). Also supporting this hypothesis, it has been shown that birds, such as ducks, feeding at wastewater treatment plants, places with a high load of clinically-relevant ARB (Pärnänen et al., 2019), carry more ARGs than avocets, turnstones or penguins, which usually feed at waters less polluted by ARB (Marcelino et al., 2019). The same results are obtained when the closest humans’ relatives, the great apes, are analysed. Notably, captive apes’ microbiomes are enriched in human-associated bacterial species and present a higher abundance of ARGs than their wild relatives (Campbell et al., 2020). This fact may reflect the interchange of bacteria (eventually carrying ARGs) between captive apes and humans, either through direct contact or though apes’ food/water supply that might eventually carry such human-associated ARB. To note that this exchange could be bidirectional, providing a possible transmission route from humans to animals and *vice versa*. In favour of this concept, it has been described that human microbiome and resistome is remodelled upon living in contact with farm animals, including the acquisition of ARGs formerly present in said animals (Sun et al., 2020). Similar findings have been reported when the presence of extended spectrum β-lactamase (ESBL)-producing *Escherichia coli* in Swedish wild gulls, was compared to isolates from humans, livestock and surface water. Notably, similar ESBL-containing plasmids were found in humans and seagulls, supporting that, as stated by the authors, the presence of ESBL-producing *E. coli* in Swedish gulls is likely a consequence of anthropic pollution (Atterby et al., 2017).

To sum up, available information indicates that anthropic pollution by ARB and the ARGs they contain is a main driver for the acquisition of AR by wildlife and that the abundance of ARGs in the microbiomes of these hosts might reflect such pollution. This concept agrees with the finding that the abundance of ARGs in anthropogenically impacted environments correlates with human faecal pollution (and hence contamination by human commensals and pathogens carrying ARGs) (Karkman et al., 2019).

However, these facts do not mean that the situation is not of concern. Although wildlife is not expected to play a fundamental role in the emergence of AR, which mainly occurs in ecosystems containing antibiotics at selective concentrations and human-linked microbiomes -as the treated patients themselves (Hernando-Amado et al., 2019)- it can be still relevant for AR transmission (**Figure 1**). Indeed, once ARB have been acquired by wildlife hosts, they can be transmitted to other environments, humans included (Sun et al., 2020), and transported through different geographic areas, hence contributing to AR spread locally and worldwide.

## The Role of Wildlife in the Origin and the Horizontal Transfer of Antibiotic Resistance

The clinical problem of AR derives from the recent -in evolutionary terms- emergence and spread of ARB and ARGs as a consequence of the use of antibiotics to treat bacterial infections (Martínez, 2012). Consistent with the fact that wild animals are not treated with antibiotics, there are no clear evidences of the emergence of AR in wildlife in recent times. Nonetheless, this does not mean that clinically-relevant AR did not evolve before the antibiotics’ era, since ARGs are ancient elements that evolved before the clinical use of antibiotics (Martinez, 2008). Indeed, intrinsic AR genes are present in all organisms (Fajardo et al., 2008; Cox and Wright, 2013; Olivares et al., 2013). Consequently, nearly any ecosystem and microbiome, including the human gut (Ruppé et al., 2019), can be a source of ARGs (Martinez et al., 2015a; Martinez et al., 2015b). Besides intrinsic AR, some evidences suggest that some mobile ARGs of clinical relevance might have been acquired in the wild as a consequence of selective pressure before the antibiotic era. In fact, a recent study shows that some specific lineages of methicillin-resistant *Staphylococcus aureus* (MRSA) appeared in hedgehogs in the pre-antibiotic era (**Figure 1**). Notably, the hedgehog dermatophyte *Trichophyton erinacei* produces two β-lactams that might be the selective force underlying MRSA selection in these mammals (Larsen et al., 2022).

Besides contributing to the origin of some ARGs in the pre-antibiotic era, as nearly any ecosystem (Martinez et al., 2007; Martinez, 2008), wild animals might also be contributing to the dissemination of recently acquired ARGs among bacteria present in their microbiomes. This possibility has been experimentally demonstrated using cockroaches (Anacarso et al., 2016), which are ubiquitously distributed and are particularly linked to human environments, including homes, farms and hospitals where AR transmission is rampant. Further, the detailed analysis of the microbiome and the resistome of *Blattella germanica* populations fed with added faeces to their diet showed the presence of mobile elements and ARGs in their metagenomes (Domínguez-Santos et al., 2021). Furthermore, it has been described that those cockroaches captured in health care facilities present in their gut a high prevalence of the pathogens circulating in said facilities (Mehainaoui et al., 2021). Other animals co-existing with humans, such as rats, flies or fleas, known to be involved in the transmission of life-threatening infectious diseases, might also contribute to the horizontal transfer of ARGs among different pathogens. Indeed, the analysis of the microbiome of *Rattus norvegicus* from hospital environments showed a high abundance, as compared with other environments, of vancomycin resistance genes linked to the transposon Tn*1549* (Hansen et al., 2016). Moreover, the use of rats, mice or flies as models has shown that ARGs can be efficiently transferred among members of the microbiomes of these animals (Jacobsen et al., 2007; Akhtar et al., 2009; Bakkeren et al., 2019; Thumu and Halami, 2019). In the case of fleas, the vector of *Yersinia pestis*, which has caused the deaths of millions of people (Achtman et al., 1999), it has been found that the growth of this bacterium in the flea’s midgut induces high-frequency conjugative genetic exchange, making then fleas not just the needed vector for plague spread but also a good environment for the acquisition, through horizontal gene transfer (HGT), of ARGs by this deadly pathogen (Hinnebusch et al., 2002). All these results support that, besides playing a role in the transmission among hosts of ARB, wild animals can also be involved in the dissemination of ARGs through HGT among different bacteria, which can later on disseminate among different hosts. Also supporting this concept, it has been described that plasmids -frequently similar to those found in human pathogens- carrying ESBLs, and eventually contributing to the HGT of these ARGs, are found in bacteria from a variety of wild animals (Smet et al., 2010; Dolejska and Papagiannitsis, 2018; Guyomard-Rabenirina et al., 2020).

Despite these observations, the role that wild animals may have in the spread, through HGT, of ARGs among bacteria -including human pathogens- which can be present in their microbiome, particularly in habitats with a high AR prevalence -i. e. hospitals, farms or wastewater-, remains largely unexplored.

## Local Transmission of Antibiotic Resistance Through Wildlife

The study of AR transmission has been mainly focused on health care facilities, places with crowded human populations, which are more prone to infections than healthy people and hence more likely to receive antibiotic treatment, the main force behind AR selection. While hospitals, frequently presenting circulating, recalcitrant antibiotic-resistant clones, are certainly the main place for the origin, evolution and spread of AR; other reservoirs, mainly farm animals, food and wastewater, have also been explored as relevant elements in the dissemination of AR among different, yet interconnected, ecosystems (Baquero et al., 2019; Hernando-Amado et al., 2019). Wildlife can also have a role in this transmission at two levels: local transmission -a One Health problem- and transmission between geographically distant areas -a Global Health issue- (**Figure 1**).

It is important to notice that wildlife does not necessarily mean human-disconnected life. As mentioned above, cockroaches, wild insects regularly present in human habitats, might play an important role, as bioreactors, allowing the dissemination of ARGs among human pathogens (**Figure 1**). Besides, they can also contribute to the maintenance of ARGs and ARB in the long term. Flies are also close to humans. It has been found that these insects can capture antibiotic-resistant human pathogens when feeding and that this situation might enhance their spread, since these bacteria replicate in the digestive tract, mouthparts and regurgitation spots of the flies (Onwugamba et al., 2018). These results imply that certain insects, such as cockroaches or flies, may serve as vectors of AR dissemination among interconnected environments; as above stated, a One Health issue. Other wild animals that are regularly present in human-linked habitats are rats. Different studies have shown the presence in their guts of ARGs with relevance for human health as ESBLs (Guenther et al., 2013; Hoang et al., 2020), frequently found in human commensals or bacterial pathogens. This fact means that rat faeces, eventually present on fomites, can be a source for the transmission of ARGs and ARB.

Something similar may apply to other non-migrating animals that do not always present close contact with humans. The analysis of the microbiome of several wild animals -iguanas, birds, anoles and rodents- from Guadeloupe showed that all of them contained ARGs also found in humans, being the percentage higher in rodents (Guyomard-Rabenirina et al., 2020). Albeit, while flies and cockroaches are frequently and inadvertently in contact with humans, it is debatable if wild animals, such as iguanas or anoles, not so frequently in direct contact with humans, might have a relevant role in AR dissemination.

The role of wildlife in AR dissemination is not restricted to the human domain. For instance, flies and cockroaches present in farms might be, in addition to human-animal contacts or sludge reuse, a relevant link between animal farms and urban human communities, allowing the transmission of ARB between these two different ecosystems (Zurek and Ghosh, 2014). Further, it has been suggested that vultures and other scavengers that feed on livestock carcasses might ingest ARB if they are present in such carcasses (Blanco and Bautista, 2020). Besides, bats, feeding on alive farm animals, contain in their microbiomes *E. coli* isolates belonging to the most prevalent sequence types causing infections worldwide, as well as several of the ESBLs that are of particular concern at hospitals (Benavides et al., 2022).

Overall, these studies support that wild animals might provide a link between two of the reservoirs that have been classically considered as the main drivers of AR: humans and farm animals (Aarestrup, 2005). In this regard, wildlife can provide a bridge that allows AR transfer among disconnected ecosystems, a feature also of relevance for animals that regularly travel long distances, as discussed below.

## Long Distance Transmission of Antibiotic Resistance Through Wildlife

Several wild animals do not travel long distances, hence contributing just to the local dissemination of AR. A different matter refers to migrating animals (**Figure 1**), particularly birds that can travel long distances even between different continents (Zeballos-Gross et al., 2021). Various studies have shown that the microbiomes of wild birds frequently contain human bacterial pathogens, including clones prevalent at hospitals, as well as ARB (Oteo et al., 2018; Stępień-Pyśniak et al., 2019; Dec et al., 2020). Further, they also carry ARGs of concern at hospitals, and these genes are frequently located in the same plasmids that cause the spread of AR among human populations (Atterby et al., 2017; Zhang et al., 2021). It is important to note that some species of gulls are well adapted to anthropogenically-influenced environments (Alexander, 2016), including habitats receiving wastewater effluents (Weiser and Powell, 2010), where clinically relevant ARGs and ARB are present. This ecological behaviour allows these birds to acquire ARB from human inputs (Varela et al., 2015). Upon migration, these birds can become vectors for the dissemination, mainly through their excreta, of clinically-relevant ARGs and ARB between distant geographical areas; as above stated, a Global Health issue. In support of this concept is the finding of AR plasmids in samples from Arctic birds, a place with low (if any) antibiotic pollution (Sjölund et al., 2008) and scarce human population. Further, a recent analysis based on the combination of biological -phenotypic and genotypic- and satellite telemetry approaches to track gull movements has shown that these birds can acquire ARB from anthropogenic sources. Later on, these birds allow ARB intercontinental spread *via* migratory movements (Ahlstrom et al., 2021).

Most studies in the field have focused on migratory birds as vectors for long-distance AR dissemination. However, aquatic animals also travel long distances and hence might also participate in such spread. In this respect, it is worth stating that water-dwelling bacteria are the origin of some predominant ARGs such as members of the *qnr, fos* or *oxa* families (Poirel et al., 2005; Potron et al., 2011; Ortiz de la Rosa et al., 2022), indicating that water organisms might also be reservoirs of AR. Most works on the role of aquatic ecosystems in selection of ARGs and ARB have focused on fish farms, because antibiotics, regularly used in these places, can select ARB (Cabello et al., 2013). Nevertheless, free-living animals might also contribute to the dissemination of AR through water bodies. The analysis of aquatic animals at coastal waters showed that they contain a variety of ARGs conferring resistance to sulphonamides, tetracycline or chloramphenicol, despite these antibiotics were not present in such waters (Hong et al., 2018). These reports support that ARGs can persist in aquatic animals even in the absence of antibiotics. These ARGs were found, not just in the gut, but also in the skin of these animals, indicating the possibility of their transfer through the food chain.

Also supporting the role of aquatic ecosystems in AR spread, sea turtles have been proposed as sentinels for AR dissemination in water ecosystems (Alduina et al., 2020). In favour of this situation is the finding of several ARGs and ARB in *Caretta caretta* turtles form the Mediterranean Sea (Trotta et al., 2021). It is expected that, as in the case of birds, turtles acquire ARB while feeding at their reproduction site, and antibiotic resistance may spread such resistant bacteria and the ARGs that they contain, through defecation among other geographical areas.

Several evidences support that wild animals can transport in their microbiome ARGs and ARB across long distances. However, the extent to which this transmission is an important step forward in AR dissemination or it has just an incremental role in such spread taking into consideration other ways of long-distance transmission -i.e. human travelling or transportation of food, farm animals or pets- is a feature that has not been explored in detail yet.

## Concluding Remarks

It is generally accepted that AR is a One Health and Global Health problem in which different ecosystems and geographical areas are involved in its emergence, evolution and spread. Most studies in the field are based on the environments closest to humans and, among them, wastewater, farm animals and pets have received particular attention. Current evidence supports that the same ARB and ARGs causing problems in humans can also be found in wildlife, and that their prevalence is higher in areas with a higher human presence; indicating that AR in wildlife is a sign of anthropic pollution. In addition, wild organisms can contribute to AR evolution and dissemination. Some be reservoirs and vectors for the local dissemination of AR. Besides, migrating animals might be contributing to AR dissemination across distant geographic areas. However, despite of these findings, quantitative analyses concerning the relevance of wildlife pathways in AR dissemination vs other ones -i. e. human travellers, vs migratory birds; or fomites and direct human-to-human contact, vs flies and cockroaches co-existing with humans- remain to be performed. In the current situation, in which climate change may alter the worldwide distribution of wildlife, including vectors involved in the dissemination of infectious diseases (Agache et al., 2022), quantitative information concerning wildlife’s role in the origin, evolution and transmission of AR is relevant to tackle AR from One Health and Global Health perspectives.

## Author Contributions

All authors listed have made a substantial, direct, and intellectual contribution to the work, have participate in writing the draft and the final version of the review, and approved it for publication.

## Funding

Work in our laboratory is supported by Instituto de Salud Carlos III (grant RD16/0016/0011) - cofinanced by the European Development Regional Fund “A Way to Achieve Europe”, by grant S2017/BMD-3691 InGEMICS-CM, funded by Comunidad de Madrid (Spain) and European Structural and Investment Funds and by MCIN/AEI/10.13039/501100011033 (PID2020-113521RB-I00). PL is recipient of a FPU fellowship and TG-G of a FPI fellowship, both from MINECO. LO-S is supported by a postdoctoral fellowship from Consejo Nacional de Ciencia y Tecnología (CONACyT-México).

## Conflict of Interest

The authors declare that the research was conducted in the absence of any commercial or financial relationships that could be construed as a potential conflict of interest.

## Publisher’s Note

All claims expressed in this article are solely those of the authors and do not necessarily represent those of their affiliated organizations, or those of the publisher, the editors and the reviewers. Any product that may be evaluated in this article, or claim that may be made by its manufacturer, is not guaranteed or endorsed by the publisher.
